# Effect of Arabic Qahwa on Blood Pressure in Patients with Stage One Hypertension in the Eastern Region of Saudi Arabia

**DOI:** 10.3390/jpm13061011

**Published:** 2023-06-18

**Authors:** Sahbanathul Missiriya Jalal, Saad Hamoud Alsebeiy, Hamida Ali Aleid, Sukinah Ali Alhamad

**Affiliations:** 1 Department of Nursing, College of Applied Medical Sciences, King Faisal University, Al-Ahsa 31982, Saudi Arabia; 2 Primary Health Centre, Health Cluster, Al-Ahsa 31982, Saudi Arabia; 3 Prince Saud bin Jalawy Hospital, Al Mubarraz 36424, Saudi Arabia

**Keywords:** hypertension, systolic blood pressure, diastolic blood pressure, Arabic Qahwa, Arabic coffee, Saudi coffee, lipid profile

## Abstract

Hypertension (HT), which is a condition of increased blood pressure (BP), is a major health problem globally. In Saudi Arabia, morbidity and mortality rates are increasing due to HT. Arabic Qahwa (AQ) is a commonly consumed beverage in Saudi Arabia and has numerous health benefits. We conducted a randomized control trial to investigate the effect of AQ on BP among patients with HT (Stage 1). Based on the inclusion criteria, 140 patients were randomly selected, and 126 patients were followed up. After obtaining demographic information, we assessed the BP, heart rate, and lipid profile before and after the intervention of consuming four cups of AQ daily for four weeks. A paired ‘t’ test was used with a significance level of 5%. In the AQ group, there were significant changes (*p* = 0.009) in systolic blood pressure (SBP), with means of 134.72 ± 3.23 and 133.14 ± 3.69 observed pre and post-test, respectively. Similarly, the diastolic blood pressure (DBP) pre- and post-test mean scores were 87.08 ± 1.8 and 85.98 ± 1.95, respectively, which also showed significance (*p* = 0.001). There were also significant changes (*p* = 0.001) observed in the lipid profile of the AQ group. In conclusion, AQ is effective in reducing SBP and DBP in patients with stage one HT.

## 1. Introduction

Hypertension (HT) is a chronic condition involving increased blood pressure (BP) and contributes to many serious health problems including heart disease, coronary artery disease, peripheral artery disease, kidney disease, and cerebro-vascular disease [1,2]. HT is a global burden of disease that leads to high morbidity and mortality rates [3]. The HT prevalence is significantly increasing due to lifestyle modifications [4] such as changing dietary patterns, lack of exercise, stress, drinking alcohol, and smoking [5]. Certain chronic illnesses such as diabetes mellitus, kidney diseases and sleep apnea due to inadequate sleep increase the risk of developing HT. Globally, HT [6,7] causes around 54% of stroke cases and 47% of heart disease cases.

In Saudi Arabia, according to the statistical evidence, there is a high prevalence of HT. According to 2010 estimates, HT was the leading risk factor for death in Saudi Arabia [8], and its control and prevention necessitate serious measures [9,10]. The Seventh Report of the Joint National Committee on Prevention, Detection, Evaluation, and Treatment of High Blood Pressure defines stage one HT as a systolic BP ranging from 130 to 139 mm Hg and a diastolic BP ranging from 80 to 89 mm Hg [11]. Pharmacological therapy and non-pharmacological management are important for reducing BP and controlling HT, which could otherwise lead to complications such as stroke [12,13].

Arabic Qahwa (AQ) is a popular Saudi coffee (Arabic coffee) and the most traditional drink in Middle Eastern countries, including Saudi Arabia. The annual consumption of AQ exceeds 400 billion cups [14] and has increased four percent per year between 2016 and 2021. It is also forecast to rise five percent by the year 2026, according to a report by global business analysts Euromonitor International. Furthermore, consumption of AQ is expected to reach 28,700 tons per year [15]. AQ is made from lightly roasted coffee beans combined with a mixture of fragrant spices such as cardamom, ginger, cloves, and saffron. Traditional AQ is usually unsweetened. It is rich in vitamins such as B1, B3 and B5 [16], which can increase the immunity of the body [17]. AQ also contains two fat soluble constituents, namely, cafestol and kahweol, which are significant constituents of coffee [18].

The ingredients of AQ contain phytonutrients, which act as antioxidants [19]. The antioxidants have benefits for reducing the risk of heart problems, such as coronary heart disease. In addition, the antioxidative status reduces low-density lipoprotein oxidation. AQ has a low sodium concentration and high potassium. This helps to hydrate the body. Hence, it may help in the management of BP [20]. AQ contains spices such as cardamom, ginger, cloves, and saffron. The cardamom in AQ promotes anti-inflammatory action, lowers blood pressure and protects against the growth of cancer cells. Research has proved that cardamom effectively lowers the blood pressure, enhances fibrinolysis, and improves antioxidant status among individuals with stage one hypertension [21].

Arabic coffee is ingrained in the culture of Middle Eastern countries. Drinking it is the tradition and cultural habit of people living in the Arabian Peninsula. AQ is widely consumed in the Arabic regions. It is usually served with dates, candied fruit, dried fruit, or nuts. Some people usually drink it with chocolates, while others are used to drinking AQ without any sweeteners.

Even though AQ has numerous health benefits, research on its effect on human health is scarce. A few studies have been conducted to investigate the causal relationship between AQ and BP among healthy subjects. However, no studies are available regarding the impact of AQ on HT patients. Therefore, we aimed to determine the effect of drinking AQ on reducing BP among patients with stage one HT, and the lipid profile of the patients was measured to investigate the mechanism of AQ’s effects.

## 2. Materials and Methods

### 2.1. Study Design

A randomized control trial was conducted to determine the effect of AQ on BP among patients with stage one HT in selected primary health centers in the eastern region of Saudi Arabia. This research was carried out from May 2022 to September 2022. The objective and procedures performed in the study were fully understood by the participants, and all of them provided informed consent for inclusion in the study. The protocol of the research was approved by the Institutional Review Board of King Faisal University, in Al-Ahsa, in the Eastern Province of the Kingdom of Saudi Arabia. This research was conducted in accordance with the Declaration of Helsinki and followed ethical principles.

### 2.2. Participants

**Figure 1:**
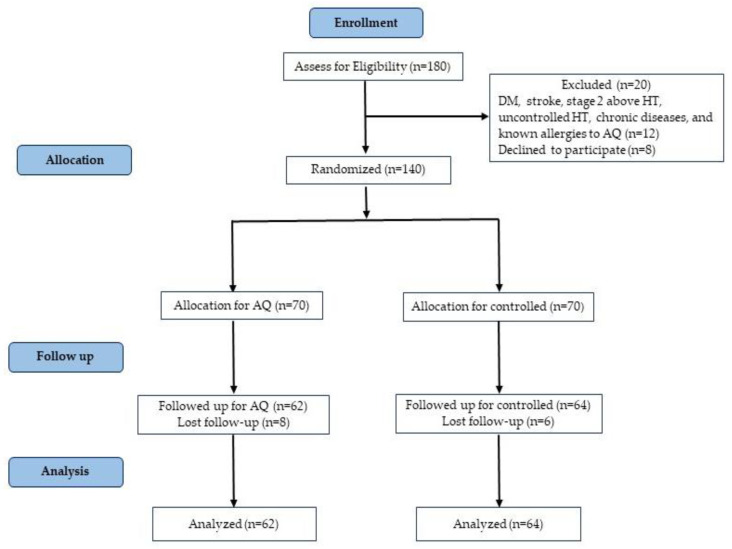
Consort flow diagram.

The inclusion criteria were patients with stage one HT, aged 20 to 59 years, including both male and female genders residing in the eastern region, with systolic BP ranging from 130 to 139 mm Hg and diastolic BP ranging from 80 to 89 mm Hg. People who had the habit of drinking AQ occasionally were included in the study. Patients with diabetes mellitus, stroke, high BP, uncontrolled HT, chronic illnesses such as cardiovascular diseases and renal diseases, those not interested in participating in the study, and those with known allergies to AQ were excluded. Considering the mean and standard deviation of a similar previous study [1], the required sample size was calculated as 139, which was rounded up to 140. Figure 1 shows that the study participants were randomly divided equally into two groups (70 in the AQ group and 70 in the control group) using a computer-generated randomized allocation with the assistance of random allocation software. Firstly, we collected the baseline data. The pre-test was followed by the intervention, which was given to the AQ group for four weeks. After the intervention period, only 126 participants (62 in the AQ group and 64 in the control group) were able to be followed up for analysis.

### 2.3. Data Collection

The participants completed a compliance questionnaire to determine self-reported adherence along with an information sheet. Initially, the demographic information was collected by a questionnaire method. The clinical information was obtained before and after the four weeks of intervention. The collected data were encrypted and stored in a secure place to ensure the privacy and confidentiality of the study subjects.

#### 2.3.1. Demographic Measures

The demographic measures were age, gender, educational level, employment status, marital status, dietary pattern, family history of HT and BMI. This baseline information was collected before the pre-test.

#### 2.3.2. Clinical Parameters

For the clinical parameters, both systolic blood pressure (SBP) and diastolic blood pressure (DBP) were assessed using an electronic BP monitor in a sitting position once per week and documented. To ensure accuracy, errors were prevented by assessing BP only in a sitting position, choosing the appropriate cuff size, properly preparing the patients, and asking the patients to refrain from speaking during measurements. The reliability of the instrument was strictly monitored. The data of SBP, DBP and heart rate (HR) before and after the four weeks of intervention were used for analysis in the study.

#### 2.3.3. Biochemical Parameters

The participants were asked to fast for 8–10 h in the morning before blood samples were collected to test their lipid profile. The levels of triglycerides, low density lipoprotein (LDL) and high-density lipoprotein (HDL) were tested in the laboratory during the pre- and post-intervention period for both the AQ and control groups.

### 2.4. Intervention

The AQ mainly comprised dried coffee beans and cardamom powder. It was prepared using one liter of safe drinking water, three tablespoons of coffee powder, one teaspoon of cardamom powder, a half-teaspoon of ground cloves, and a quarter teaspoon of ground saffron. It was allowed to boil for 15 min over a medium flame and was stored in a flask for a minimum of 10 min. Later, the clear part of the 30 mL of AQ was served to drink along with fresh dates each time. The study participants were instructed and encouraged to consume it four times daily for four weeks. The post-test was carried out after four weeks of intervention. The control group was instructed to avoid drinking AQ for those four weeks.

### 2.5. Outcome Measures

The outcome measures in this study were changes in SBP and DBP levels. The lipid profile of the patients was assessed by measuring triglycerides, LDL and HDL. A well-structured questionnaire was used to collect the data on the participants’ socio-demographic characteristics.

### 2.6. Statistical Methods

A statistical analysis was performed using SPSS 25.0 software (SPSS, Inc., Chicago, IL, USA). The results were presented as the mean ± standard deviation for continuous variables. Numbers and percentage values were used in descriptive analysis to interpret the BMI and other variables. A paired ‘t’-test was used to compare data before intervention and after intervention within the groups. The level of statistical significance was set at 0.05 to test the normality. One-way ANOVA was used to assess the association of SBP and DBP with demographic variables.

## 3. Results

### 3.1. Demographic Charecteristics

In total, 126 patients with HT participated in this study, of whom 62 formed the AQ group and 64 formed the control group. Table 1 summarizes the demographic characteristics of patients with HT. Among all those with stage 1 HT, 45% were aged between 41 and 50 years in both the AQ and control groups. The mean age in the AQ group was 46.3 ± 8.01, and the mean age in the control group was 45.6 ± 8.01. Around 55% of the participants in the AQ group were male and 45% were female. Most of them (97% in the AQ and 92% in the control group) were married. Regarding diet patterns, approximately, 79% in the AQ group and 70% in the control group preferred a non-vegetarian diet. Regarding family history of illnesses, 43% in the AQ group and 41% in the control group presented risks of HT. No differences were identified between the AQ and control groups in age, gender, educational level, occupation, marital status, diet pattern, or family history of HT.

### 3.2. Anthropometric Measures

**Figure 2:**
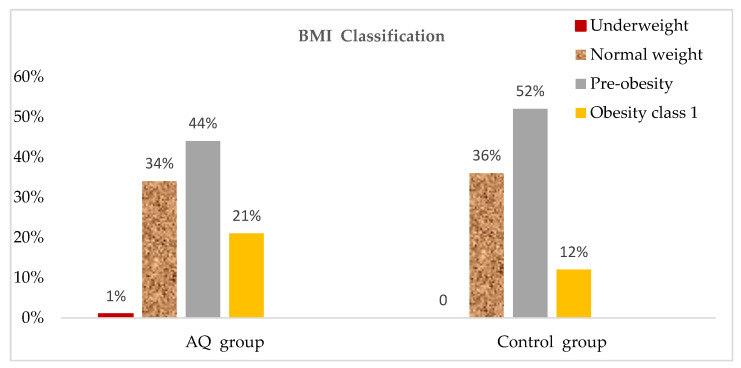
Frequency distribution of BMI.

The frequency distribution of BMI status is shown in Figure 2. Around half of the participants of each group were in pre-obesity condition, and 21% of the AQ group were obesity class one status. Around 34% in the AQ group, and 36% in the control group had normal weight.

### 3.3. Clinical Measures

In the AQ group, there were significant changes observed in SBP. These were 134.72 ± 3.23 and 133.14 ± 3.69 pre- and post-test, respectively, which were statistically significant differences (*p* = 0.009). Similarly, the DBP pre- and post-test results showed that the mean scores were 87.08 ± 1.8 and 85.98 ± 1.95, respectively, which were also significant (*p* = 0.0001). The paired ‘t’ test results (Table 2) showed that AQ was effective in reducing both SBP and DBP. However, there were no statistically significant changes observed in the control group. Regarding HR, there were no significant variations observed in either the AQ or control group.

### 3.4. Biochemical Measures

A paired ‘t’-test was used with at a significance level of 5%. Table 3 shows that, in the AQ group, the triglyceride level was 115.28 ± 9.72 in the pre-test and 113.25 ± 10.2 in the post-test, which indicated a slight decrease, with high significance (*p* = 0.0001). The mean LDL was 99.95 ± 3.41 and 98.76 ± 3.27 in the pre- and post-tests, respectively, which decreased slightly, with high significance (*p* = 0.0001). Similarly, the mean HDL level was 42.35 ± 5.003 in the pre-test and 53.45 ± 3.4 in the post-test, showing a good increase in HDL, with high significance (*p* = 0.0001). There was a positive correlation observed between SBP and DBP in the AQ group in the post-test value (R = 0.5754; *p* = 0.0001). Regarding the biochemical parameters, a strong positive correlation was observed (R = 0.8615; *p* = 0.0001) between triglycerides and LDL in the AQ group. However, a moderately negative correlation was found (R = −0.7198; *p* = 0.0001) between LDL and HDL.

### 3.5. Association of BP and Demographic Variables

The association between the SBP and DBP of the AQ group before intervention with the selected demographic variables is shown in Table 4. A significant association was found between SBP and the family history of HT (*p* = 0.0412) and between DBP and the family history of HT (*p* = 0.0428). Otherwise, no other significant associations were identified between SBP and DBP and any other demographic characteristics.

**Figure 3:**
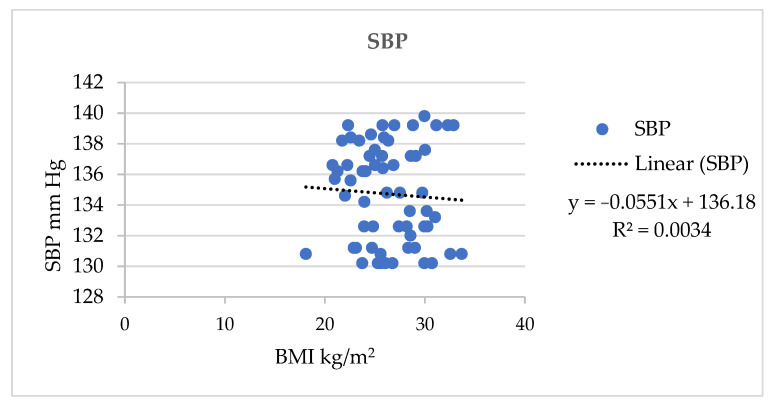
Association of SBP and BMI in in the AQ group.

There was a significant (*p* = 0.001) association found between BMI and SBP in the AQ group which is shown Figure 3.

**Figure 4:**
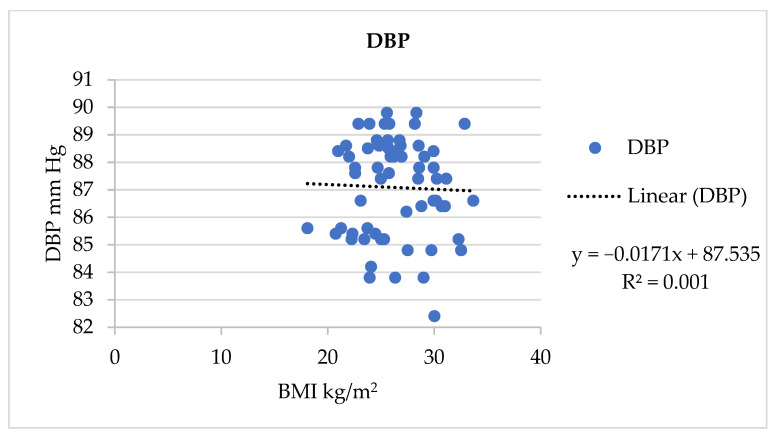
Association of DBP and BMI in in the AQ group.

Similarly, a significant association (*p* = 0.001) was found between BMI and DBP in the AQ group, which is shown in Figure 4.

## 4. Discussion

HT is a major cause of premature death worldwide, and one of the global targets for noncommunicable diseases is to reduce its prevalence by 33% between 2010 and 2030 (WHO) [22]. There are many ways to achieve this reduction [23], and self-care management is one of the key ways. It is essential to follow lifestyle modifications in areas such as diet, physical exercise, and relaxation techniques and to avoid or quit bad social habits. Many people use nutritional therapy or homemade food to reduce the severity of HT. One such method is the consumption of Arabic coffee which helps to reduce BP. AQ is known as a Saudi coffee and it has become one of the most popular drinks in the world. It is widely used in Arab countries, but people in more than 50 countries are currently growing and consuming this coffee.

Most studies have investigated the association between coffee consumption and BP [24,25,26] among healthy people. However, there is a lack of research on the effect of AQ on BP among people with HT. Therefore, a randomized controlled trial was conducted to determine the effect of AQ on BP (including SBP and DBP) among patients with stage one HT. Significant changes (*p* = 0.009) were observed in the SBP of the AQ group, which was 134.72 ± 3.23 and 133.14 ± 3.69 in pre- and post-test, respectively. Similarly, the mean score of DBP among the AQ group in the pre- and post-tests was 87.08 ± 1.8 and 85.98 ± 1.95 respectively, which also showed high significance (*p* = 0.0001). These findings are supported by a study conducted to test the effect of Arabian coffee (Saudi coffee) consumption on BMI, blood glucose level, and BP, which concluded that Saudi coffee lowers all three [27]. However, another study conducted among healthy women aged between 18 and 40 years to identify the dose-dependent effects of coffee with or without two doses of cardamom, showed that the consumption of Arabic coffee either with or without cardamom, did not alter BP, as assessed by a paired comparison ‘t’ test [28].

A study in Addis Ababa investigating non-diabetic individuals tested the effect of habitual consumption of Ethiopian Arabica coffee on the risk of cardiovascular diseases. The tool used for data collection consisted of demographic variables, obtained using structured questionnaires and anthropometric measurements measured according to the WHO standards. The study results evidenced that the regular consumption of Ethiopian Arabic coffee significantly increased serum high density lipoprotein cholesterol and significantly decreased triglyceride levels in the blood serum [29]. In our study, we followed the same method for the data collection of demographic variables. The anthropometric measurements were calculated using Quetelet’s index as per WHO recommendations. In the AQ group, the triglyceride level showed a slight decrease in the post-test after intervention, with high significance (*p* = 0.0001). Similarly, the mean HDL level was increased in the post-test after intervention. The level of LDL decreased slightly, indicating high significance (*p* = 0.0001) in the AQ group. Other research on the effect of drinking normal coffee consumption on lipid levels showed that there were no significant effects [30]. The difference between normal coffee and AQ has been scientifically analyzed. The coffee beans are usually more roasted in normal coffee with the result that the roasted beans contain reduced amounts of caffeine and water content, whereas in AQ coffee, the beans are lightly roasted and mixed with cardamom, and sometimes cloves, saffron or ginger. In addition, a difference in LDL was not observed in another study [29]. This research investigated the relationship between coffee consumption and serum lipids. The results indicated that the consumption of unfiltered coffee significantly contributed to increases in TC, LDL-C and triglycerides, and these changes were related to the level of consumption [31].

One epidemiological study found an association between usual coffee consumption and obesity based on BMI and waist circumference, but other results in the literature are inconsistent. Some studies found that coffee consumption was effective in decreasing body weight and BMI in preventing obesity [32], while other studies reported that increased coffee consumption was associated with an increase in BMI and waist circumference [33]. Several other studies did not identify any association between coffee consumption and the risk of obesity [34].

In the present study, the frequency distribution of BMI status (Figure 2) showed that nearly 50% of the participants in each group were in a pre-obesity condition. Furthermore, 20.97% of the AQ group were obesity class one status. Around 33.87% had a normal BMI. A significant association was found between BMI and BP. A study conducted to assess the heterogeneity of the association between BMI and BP across a wide variety of subgroups of the Chinese population supported our study’s results by proving that BMI was positively associated with BP [35]. As BMI increases, SBP and DBP also increase. This was highlighted with linear regression in Figure 3 and Figure 4, respectively.

HT is one of the contributors to the burden of non-communicable disease around the world. Family history is an important non-modifiable risk factor for chronic illnesses such as HT. A study was conducted in Asia among a nationally representative sample from Sri Lanka. This study aimed to identify the influence of family history on the prevalence of HT and associated metabolic risk factors among a large cohort of South Asian adults. The results proved that there was an influence of family history of HT on disease prevalence and associated metabolic risk factors among Sri Lankan adults, as the prevalence of HT was significantly higher in those with a family history of HT [36]. We observed that a significant association was found between SBP and the family history of HT (*p* = 0.0412) and between DBP and family history of HT (*p* = 0.0428) in our study. 

Several research studies have been conducted in various regions of the Kingdom of Saudi Arabia regarding the consumption of coffee among the general population and the risks associated with drinking coffee. However, there are only a few studies available specifically about the effects of Arabic coffee. A cross-sectional study conducted in the city of Madinah, Kingdom of Saudi Arabia aimed to investigate the effects of drinking Arabic coffee on obesity among 384 females. The study found that those females who drank excessive Arabic coffee accompanied by additives and sweets had an increased risk of obesity [37].

Another study examined Saudi coffee consumption and its association with obesity among the Saudi general population living in the Eastern Province. The study results showed that frequent and excessive consumption of Arabic coffee was predicted to have a direct association with obesity in females, specifically when it was mixed with calorie containing additives such as milk, cardamom, and other additives. Furthermore, drinking Arabic coffee increased the risk of obesity when it was taken with additives along with eating chocolate and dates [38]. A study conducted in Korea revealed a similar relationship between the obesity of women and the consumption of coffee. A multiple logistic regression model was used to analyze the odds ratio in the study. The findings were that there was a positive correlation between the amount of coffee with additives consumed daily by Korean women and the prevalence of obesity. However, due to the study design, which was based on a cross-sectional method, the causative factors could not be determined [39].

Another study also found that the increase in BMI level was due to the additives and food consumed with Arabic coffee [40]. A similar study was conducted among the Saudi female population, in which they identified that high levels of daily coffee intake were directly associated with obesity among regular coffee drinkers [41,42]. Another study was performed to identify the association between BMI and waist circumference and the use of additives in coffee or tea. It was found that the frequency of coffee or tea consumption was not associated with measures of obesity. Moreover, the use of artificial sweeteners within coffee or tea was associated with higher BMI compared with those who did not use them [43].

In the present study, the participants consumed AQ with dates. Some researchers have identified that the date fruit has great nutritional value, and some studies have evidenced that two to three servings of date fruit daily can be beneficial for patients with diabetes mellitus, as it has a positive effect on glycemic control [44]. In the current study, the glycemic index of participants was not measured as those with diabetes mellitus were excluded.

Nevertheless, the current study has several strengths, such as randomization, and the inclusion of participants of both genders with different age groups, different body compositions, and different diet patterns. However, a few limitations were also identified. We could not understand the long-term effects of AQ as the intervention lasted for four weeks and the study had very limited parameters.

## 5. Conclusions

The authors of the present study conclude that Arabic Qahwa lowers SBP and DBP significantly. Qahwa is a staple food and traditional drink of Arabic people, and as this AQ is a common food beverage not only in Saudi Arabia but in all Middle Eastern countries, this finding will be very useful in controlling and managing HT at earlier stages. This beverage can be served in other countries too, as an alternative to regular coffee. Further research in the future is recommended to investigate the causal relationship between AQ and diabetes mellitus.

## Figures and Tables

**Table 1:** Demographic characteristics of patients with HT.

Demographic Characteristics		AQ (*n* = 62)	Control (*n* = 64)	*p* Value
		N (%)	N (%)	
Age	20–30 years	3 (5)	4 (6)	0.869
	31–40 years	12 (19)	15 (24)	
	41–50 years	28 (45)	29 (45)	
	51–59 years	19 (31)	16 (25)	
Gender	Male	34 (55)	32 (50)	0.587
	Female	28 (45)	32 (50)	
Educational level	Primary	4 (6)	5 (8)	0.547
	High school	14 (23)	20 (31)	
	Higher secondary	34 (55)	27(42)	
	Graduate	10 (16)	12 (19)	
Employment status	Employed	39 (63)	34 (53)	0.266
	Unemployed	23 (37)	30 (47)	
Marital status	Unmarried	2 (3)	5 (8)	0.261
	Married	60 (97)	59 (92)	
Diet pattern	Vegetarian	13 (21)	19 (30)	0.261
	Non-vegetarian	49 (79)	45 (70)	
Family history of HT	Yes	27 (43)	26 (41)	
	No	35 (57)	38 (59)	0.74

**Table 2:** Effect of AQ on level of BP.

Measurements	AQ Group (*n* = 62)			Control (*n* = 64)		
	Pre-Test Mean (SD)	Post-Test Mean (SD)	Paired ‘t’ Test	Pre-Test Mean (SD)	Post-Test Mean (SD)	Paired ‘t’ Test
SBP (mm Hg)	134.72 ± 3.23	133.14 ± 3.69	t = 8.397; *p* = 0.009 *	136.49 ± 2.97	136.72 ± 2.79	t = 0.984; *p* = 0.329 NS
DBP (mm Hg)	87.08 ± 1.8	85.98 ± 1.95	t = 9.069; *p* = 0.0001 *	87.08 ± 1.79	87.19 ± 1.92	t = 0.684; *p* = 0.497 NS
HR (bpm)	78.13 ± 5.02	77.21 ± 4.82	t = 1.8376; *p* = 0.7099 NS	78.41 ± 5.16	78.55 ± 5.25	t = 0.4695; *p* = 0.643 NS

* Significant; NS—Not significant.

**Table 3:** Lipid profile.

Measurements	AQ Group (*n* = 62)			Control (*n* = 64)		
	Pre-Test Mean (SD)	Post-Test Mean (SD)	Paired ‘t’ Test	Pre-Test Mean (SD)	Post-Test Mean (SD)	Paired ‘t’ Test
Triglyceride (mg/dL)	115.28 ± 9.72	113.25 ± 10.2	t = 7.2117; *p* = 0.0001 *	115.87 ± 10.03	115.84 ± 9.94	t = 0.0361; *p* = 0.9713 NS
LDL (mg/dL)	99.95 ± 3.41	98.76 ± 3.27	t = 6.636; *p* = 0.0001 *	99.74 ± 3.43	100.12 ± 3.997	t = 0.7717; *p* = 0.4434 NS
HDL (mg/dL)	42.35 ± 5.003	53.45 ± 3.4	t = 6.4034 *p* = 0.0001 *	42.01 ± 5.096	42.53 ± 5.24	t = 1.9404; *p* = 0.05711 NS

* Significant; NS—Not significant.

**Table 4:** Association between SBP and DBP with demographic variables.

Demographic Characteristics		AQ (*n* = 62) N (%)	SBP Mean Score	*p* Value	DBP Mean Score	*p* Value
Age	20–30 years	3 (5)	132.13	0.1621	87.3	0.1194
	31–40 years	12 (19)	131.13	NS	86.89	NS
	41–50 years	28 (45)	133.5		85.84	
	51–59 years	19 (31)	134.03		85.41	
Gender	Male	34 (55)	132.59	0.2992	85.61	0.1027
	Female	28 (45)	133.58	NS	86.43	NS
Educational Level	Primary	4 (6)	133.95	0.3637	87.55	0.1295
	High school	14 (23)	131.59	NS	86.44	NS
	Higher secondary	34 (55)	133.55		85.87	
	Graduate	10 (16)	133.58		85.08	
Employment Status	Employed	39 (63)	133.43	0.4193	86.25	0.1539
	Unemployed	23 (37)	132.64	NS	85.52	NS
Marital Status	Unmarried	2 (3)	130.7	0.3469	86.00	0.6207
	Married	60 (97)	133.22	NS	85.3	NS
Diet Pattern	Vegetarian	13 (21)	133.5	0.6936	85.49	0.3153
	Non-vegetarian	49 (79)	133.04	NS	86.11	NS
Family history of HT	Yes	27 (43)	133.5	0.0412 *	85.46	0.0428 *
	No	35 (57)	133.86		86.38	

* Significant; NS—Not significant.

## Data Availability

The data cannot be shared due to data protection regulations. Only evaluation of anonymized data is allowed according to the responsible ethics committee.

## Abbreviations

AQ	Arabic Qahwa
BMI	Body mass index
BP	Blood pressure
bpm	Beats per minute
DBP	Diastolic blood pressure
HDL	High density lipoprotein
HT	Hypertension
HR	Heart rate
IBM	International Business Machines Corporation
LDL	Low density lipoprotein
mm Hg	Millimetre mercury
MOH	Ministry of Health
NS	Non-significant
SD	Standard deviation
SPSS	Statistical Package for the Social Sciences
SBP	Systolic blood pressure
USA	United States of America
WHO	World Health Organization
